# Strengthening research and training on insecticide resistance in arthropod vectors in South America: The WINSA network

**DOI:** 10.1371/journal.pntd.0013675

**Published:** 2025-12-09

**Authors:** Vincent Corbel, Leslie Alvarez, Kartika Doerdjan-Ramoutar, Jean-Bernard Duchemin, Stephane Duchon, Luisa Figueroa, Christian R. Gonzalez, Nilsa González-Brítez, Laura Harburguer, Audrey Lenhart, José Bento Pereira Lima, Ronald Lopez, Diego Morales, Reza Niles-Robin, Martha L. Quinones, Miriam Palomino Salcedo, Gabriela Willat, Ademir J. Martins

**Affiliations:** 1 Institut de Recherche pour le Développement (IRD), MIVEGEC, CNRS, IRD, Université de Montpellier, Montpellier, France; 2 Laboratório de Biologia, Controle e Vigilância de Insetos Vetores (LBCVIV), Instituto Oswaldo Cruz (IOC), FIOCRUZ, Avenida Brasil Manguinhos, Rio de Janeiro, Brasil; 3 Núcleo Universitario Rafael Rangel, Universidad de los Andes, Trujillo, Venezuela; 4 Anton de Kom University of Suriname (AdeKUS), Leysweg, Paramaribo, Suriname; 5 Unité d’Entomologie Médicale, Institut Pasteur de la Guyane, Cayenne, French Guiana, France; 6 Arboviruses and Insect Vectors, Institut Pasteur, Paris, France; 7 Centro de Estudios de Enfermedades Endémicas y Salud Ambiental, SAIAE-MPPS, Maracay, Aragua, Venezuela; 8 Universidad de Carabobo, Naguanagua, Carabobo, Venezuela; 9 Instituto de Entomología, Facultad de Ciencias Básicas, Universidad Metropolitana de Ciencias de la Educación, Ñuñoa, Santiago, Chile; 10 Instituto de Investigaciones en Ciencias de la Salud, Universidad Nacional de Asunción, San Lorenzo, Paraguay; 11 Facultad de Ciencias Exactas y Naturales, Universidad Nacional de Asunción, San Lorenzo, Paraguay; 12 Centro de Investigaciones de Plagas e Insecticidas (CIPEIN-UNIDEF/CITEDEF/CONICET), Villa Martelli, Buenos Aires, Argentina; 13 Centers for Disease Control and Prevention, Atlanta, Georgia, United States of America; 14 Instituto Nacional de Laboratorios de Salud (INLASA), La Paz, Bolivia; 15 Instituto Nacional de Investigación en Saude Pública (INSPI), Guayaquil, Ecuador; 16 Vector Borne Diseases, Ministry of Health, Georgetown, Guyana; 17 Departamento de Salud Pública, Facultad de Medicina, Universidad Nacional de Colombia, Bogotá, Colombia; 18 National Health Institute, Chorrillos, Lima, Peru; 19 Unidad Zoonosis y Vectores, Ministerio de Salud Pública, Montevideo, Uruguay; University of Notre Dame, UNITED STATES OF AMERICA

## Abstract

The “*South American Research Network for the Surveillance and Control of Insecticide-Resistance in Arthropod Vectors*” (WINSA), established in 2024 by the IRD and FIOCRUZ with support from the US-CDC VecNet initiative and WHO–TDR, aims to coordinate research on insecticide resistance in arthropod vectors in South America, provide a platform for regional collaboration, and develop effective mitigation strategies. WINSA brings together leading technical experts representing research institutions from 14 countries and territories located in South America, the USA, and France to promote collaboration and information exchange, identify research gaps and priorities, enhance technical capacity in insecticide resistance monitoring, and support national and regional programs on vector resistance issues. This network seeks to contribute to the reduction and elimination of vector-borne diseases in South America.

## Author summary

This manuscript describes WINSA, a newly established network comprising 15 research institutions from 14 countries across South America, the USA, and France, dedicated to advancing research and monitoring of insecticide resistance in arthropod vectors. The objectives of WINSA are to raise awareness, address knowledge gaps, and strengthen research and training opportunities for vector insecticide resistance surveillance and management. The network activities aim to strengthen regional partnerships to enhance and sustain regional vector resistance research and surveillance to improve vector-borne disease prevention and control in South America.

## Introduction

Vector-borne diseases (VBDs), especially mosquito-transmitted arboviral diseases (e.g., dengue, Zika, and chikungunya), are emerging or reemerging in South America, with increasing prevalence and severity [1]. Vector ecology is highly climate-sensitive, with environmental changes influencing reproduction, survival, and geographic distribution of vectors, thereby impacting pathogen transmission [2]. Climate and environmental changes have expanded the geographic range of vectors, with mosquito vectors now occupying the broadest distribution in recorded history [3,4].

**Fig 1:**
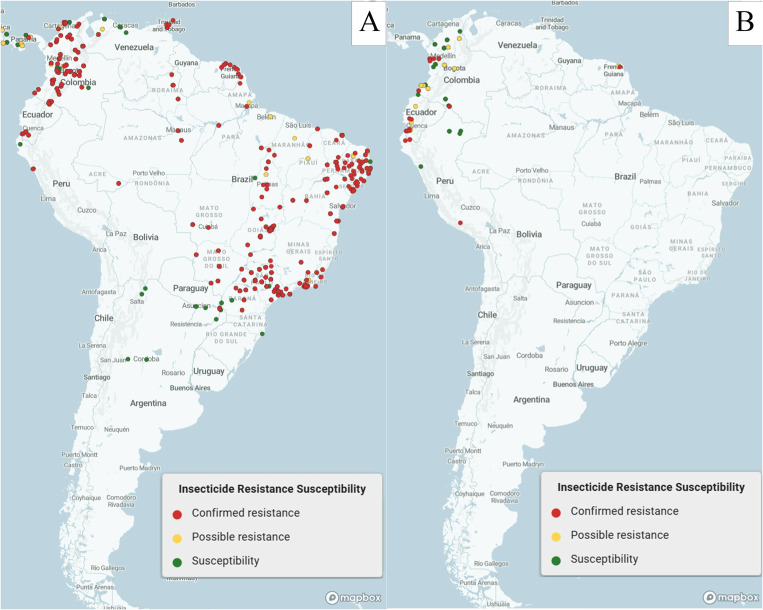
Current knowledge on insecticide resistance distribution in *Aedes spp.* (*Ae*. *aegypti* and *Ae. albopictus*, panel A, https://irmapper.com/aedes, and *Anopheles* spp. (*An. darlingi* and *An. albimanus*, panel B, https://irmapper.com/anopheles), in South America between 2000 and 2024. This represents data across all insecticides (pyrethoids, carbamates, and organophosphates) and all types of adult bioassays (CDC bottle assays and WHO filter paper tube test). The green dots indicate susceptibility (≥98% mortality), the yellow dots indicate possible resistance (mortality >90% and <98%), and the red dots indicate resistance (<90%), according to WHO guidelines [22]. IR mapper (https://www.irmapper.com/). All maps are public domain and freely available for use under a CC BY-compatible license.

In South America, vector control relies heavily on insecticide-based strategies. The region’s significant use of insecticides—a 120% increase in the last 20 years in both agricultural and public health sectors [5]—has driven the emergence of insecticide resistance, now seen across all major public health insects such as mosquitoes, sandflies, midges, and triatomines [6,7]. Based on data from formal databases (WHO threat maps and IR mapper), insecticide resistance has been reported in at least one vector insect species across all 13 countries and territories of South America. For instance, *Aedes* mosquitoes (vectors of dengue, chikungunya, and Zika) exhibit high levels of resistance to all major insecticides in the region [8] (Fig 1A). Evidence indicates an increase in resistance/tolerance among the key malaria vectors, *Anopheles albimanus* [9–11] and *An. darlingi* [12–14] (Fig 1B), while reports from leishmaniasis-endemic countries suggest pyrethroid resistance in *Lutzomyia* species in Brazil [15] and Peru [16]. Pyrethroid resistance in *Triatoma infestans* has been documented in Brazil and Venezuela [17], as well as in Bolivia [18] and Argentina [19,20]. The latter two countries have the highest incidence of Chagas disease [21]. With the exception of *Aedes* spp., there is a notable lack of accessible data documenting the prevalence and mechanisms of insecticide resistance in mosquito vector species across the South American continent, as illustrated in Fig 1, and the knowledge gaps are even greater for non-mosquito vectors.

The development of insecticide resistance in vector populations is a major threat to the success of VBD programs. Vector surveillance, including insecticide resistance, remains weak in many countries that are at risk of VBDs owing to a lack of resources, expertise, research support, and/or political will [23]. Despite its centrality to disease transmission, entomology is often not regarded as a key discipline in the control of VBDs compared to fields such as epidemiology or medicine. Consequently, it suffers from limited recognition and inadequate funding. Moreover, priorities and resources are generally directed toward the most pressing public health issues. For example, in Brazil, considerably more effort has been devoted to *Aedes* surveillance than to *Anopheles* because dengue and other arboviral diseases have posed major public health problems in the last decade [24]. In this case, the historically limited surveillance of malaria vectors reflects political decisions rather than a lack of capacity or funding. Similarly, in Argentina, extensive data on *T. infestans* (biology, ecology, and insecticide resistance) have been collected due to the historical importance of Chagas disease in the country [25,26], whereas information on other vector species remains scarce. In contrast, in countries such as Guyana, Suriname, and Venezuela, the main limitation is a lack of resources or technical capacity to conduct adequate vector surveillance, including routine insecticide resistance monitoring following standardized test protocols.

Given these challenges, the WINSA network was launched in 2024, representing a South American extension of the international WIN network [27]. WINSA aims to provide a regional platform to foster collaboration, coordinate research on shared regional priorities related to the threat of insecticide resistance, and strengthen capacity in medical entomology, with a focus on developing sustainable resistance management strategies. Support for establishing the WINSA came from IRD-WIN, CDC’s VecNet initiative, and WHO–TDR.

## Responding to a growing need

**Fig 2:**
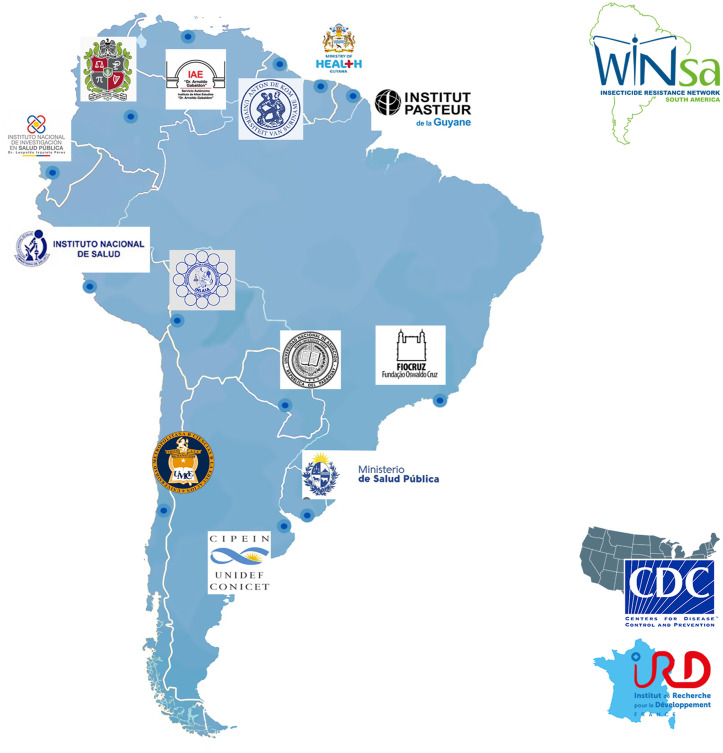
Partners of the WINSA network.

The WINSA addresses an urgent petition from South American partners for a coordinated regional approach to vector resistance research. Despite well-established research institutions, significant gaps in insecticide resistance research, monitoring, and management exist across South American countries, which was highlighted at a 2023 FIOCRUZ-sponsored meeting attended by insecticide resistance researchers from eight South American nations. The meeting identified critical resources and knowledge gaps in the field of insecticide resistance research, lack of continuity due to high staff turnover, and limited international engagement in academic and research institutions. The group also highlighted areas needing further research, including (i) biological and molecular detection methods, (ii) biology, genetics and ecology of vectors in a changing environment, and (iii) intervention research. Following this initial meeting, institutions were identified across South America, and focal points working on insecticide resistance were engaged to determine the interest in forming a regional network. Through virtual outreach, representation was confirmed across all countries and territories on the South American continent, and the WINSA was established in partnership with CDC in the USA and IRD in France (Fig 2).

## Network goals

The primary goals of the WINSA are to facilitate information-sharing, fill research gaps, develop skills, and encourage scientific collaboration to address the threat of insecticide resistance and to promote mitigation strategies. Involving academic and nonacademic stakeholders across various disciplines (entomology, molecular biology, genomics, remote sensing, and mathematics) will enable partners to access the knowledge and multidisciplinary expertise necessary for improving evidence-based vector management. WINSA will provide researchers, students, and other public health professionals with access to pertinent resources around insecticide resistance through conferences, workshops, training, educational materials, and the promotion of responsible data sharing to foster regional research collaboration.

## Materials and methods

### Governance

The WINSA governance structure comprises the following four key entities.

**1. Steering Committee (SC):** The decision-making body responsible for strategic direction, day-to-day management, and budget allocation. The committee includes two co-chairs representing the leading institutions: one from the IRD, France, and another from FIOCRUZ, Brazil.
**2. Scientific Committee (SCC):** Comprising two co-chairs and four elected members from institutions or countries different from those of the chairs, the SCC coordinates network activities, oversees the Working Groups (WGs), and reviews funding proposals. Elected members rotate annually.
**3. General Assembly (GA):** Includes all consortium members, meeting at least twice a year (with one in-person meeting minimum) to review strategies, actions, and propose improvements.
**4. Advisory Board (AB):** An optional entity of external experts providing independent advice and support for resource mobilization.

### WINSA’s four action pillars

**Fig 3:**
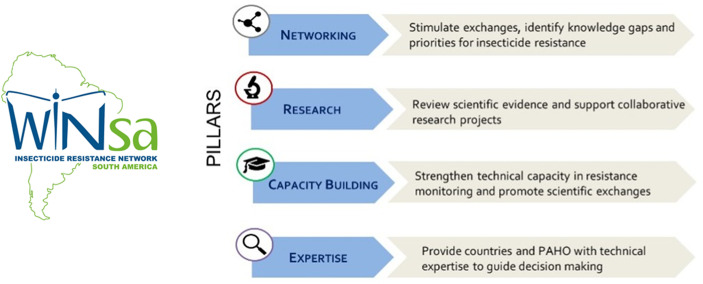
The WINSA pillars of action.

The network operates on four pillars of action: networking, research, capacity building and expertise, as illustrated in Fig 3.

#### Pillar 1: Strengthening networking among vector resistance researchers.

The planned activities include:

- Annual partner meetings to facilitate knowledge-sharing, identify gaps, and plan and coordinate the activities of the WINSA network.
- Biennial conferences to raise awareness, foster innovation, support translational research, engaging scientists, students, stakeholders, and international organizations.
- Establishment of communication networks among WINSA members to routinely share information and best practices regarding insecticide resistance research and surveillance. The network organizes monthly seminars accessible to a large audience. It also hosts a website (https://win-network-ird.fr/) and maintains a presence on social networks (i.e., https://www.linkedin.com/company/winsa-network/).

#### Pillar 2: Filling research gaps and developing resistance mitigation strategies.

The planned activities include:

- Research conducted through WGs on priority topics, such as vector distribution, resistance mechanisms, population genetics and innovative diagnostic methods.
- “Pump Prime Funding” support to initiate collaborative research projects among WINSA partners and to generate the initial evidence needed to support the submission of research proposals to national and international calls.

#### Pillar 3: Building capacity in insecticide resistance monitoring.

The planned activities include:

- Training courses for researchers, students, and program staff to strengthen capacities for monitoring insecticide resistance. Workshops are scheduled at the University of Colombia in Bogotá, Colombia (2026) for biological testing, and at FIOCRUZ in Rio de Janeiro, Brazil (2027) for molecular assays. Each workshop will host up to 15 participants and will feature a combination of scientific and technical lectures, as well as practical laboratory sessions. A “train-the-trainer” component is planned for each workshop, enabling these trainers to independently lead future sessions and thereby creating a sustainable model for continuous skill development and knowledge dissemination within the community.
- Sharing of educational resources, including manuals, protocols, and video tutorials, based on WHO-recommended testing methods, all available on the WINSA website.
- Short-term scientific exchanges (up to 15 days) to facilitate knowledge sharing, promote career development, and enhance skill acquisition among early-stage researchers. Each request will be reviewed and approved by the SCC to ensure effective use of resources and alignment with WINSA objectives.

#### Pillar 4: Strengthening expertise in vector surveillance for informed decision-making.

The planned activities include:

- Contributions to the development of an up-to-date, centralized repository of vector resistance data for South America, integrating both published and unpublished sources, to generate regional “threat maps” that can guide vector control strategies.
- Participation in national (eventually international) expert committees, offering guidance on vector control and resistance management.
- Coordination of multicenter trials to advance new testing methodologies and assess alternative control tools and strategies.

## Discussion

Widespread insecticide resistance in South America’s vector populations complicates VBD control and prevention, as seen by the recent rise in dengue, Zika, and malaria cases [28]. Limited vector surveillance capacity, including insecticide resistance monitoring, can hamper response efforts [23]. In addition, basic and applied research around insecticide resistance is often limited, restricting the availability of evidence to inform environmentally sound and effective deployment and scale up of alternative interventions—especially for new tools, technologies and approaches. For example, emerging technologies, such as the release of *Ae. aegypti* infected with *Wolbachia* for arboviral disease prevention, deployed in Brazil and Colombia, as well as the release of *Ae. aegypti* carrying a dominant lethal gene (RIDL) in Brazil, requires stringent information on the resistance genetic backgrounds of the native mosquito populations to maximize impact. Indeed, these approaches require the release of insecticide-resistant mosquitoes to survive the routine insecticide-exposure experience in the field [29]. However, there is currently no global consensus on how to deploy and monitor insecticide-resistant mosquitoes within the framework of wide-scale releases of *Wolbachia*-infected mosquitoes [30]. As a body of regional experts, WINSA would be well-placed to develop technical guidelines for guiding the release of insecticide-resistant mosquitoes in *Wolbachia* interventions and standardize post-release monitoring of resistance markers. The network is also well-positioned to conduct large-scale genomic surveillance studies, such as those on arbovirus vectors *Ae. aegypti* and *Ae. albopictus*, to investigate gene flow and migration routes as well as to identify genetic variants in loci associated with insecticide resistance and vector competence, provided adequate funding is available.

Recent advances in high-throughput barcoding [31] and MALDI-TOF [32,33] may offer promising support for vector surveillance while reducing time and cost. This is particularly relevant since insecticide resistance is increasing in South America, especially in the major malaria vectors, *An. albimanus* and *An. darlingi* [11,34], as these species face rising selection pressure from insecticides. Surveillance of malaria vector resistance should be a priority in all malaria-endemic countries in South America, including those in the process of malaria elimination and those that have already eliminated malaria. This is particularly important since the prevention of reintroduction largely relies on insecticides, through indoor residual spraying or long-lasting insecticidal nets. An urgent need exists to further investigate the relationship between insecticide resistance and vector control efficacy, including the impact of pollutants, agricultural insecticides, and domestic insecticide use on the selection of resistance to public health pesticides. Finally, there is a need to develop a framework for Insecticide Resistance Monitoring and Management in the region to provide timely and graduated responses according to the level of risk. The Integrated Plan for Insecticide Resistance Surveillance in mosquitoes, developed in France and its overseas departments (e.g., French Guiana) [35], could be adapted to offer technical guidance on implementing locally tailored surveillance and control responses based on available evidence. Beyond vector resistance, the network is committed to generating critical evidence to guide strategic approaches for vector control. For instance, WINSA has secured a $225K grant from the U.S. AMCA to conduct a multicountry randomized controlled trial evaluating the efficacy of chemical-based vector control tools against *Culicoides paraensis*, the primary vector of Oropouche virus (OROV) in South America, and assessing its insecticide resistance status. This initiative will generate essential data to inform both national and regional OROV control strategies, while also strengthening local capacity through comprehensive training of municipalities in midge collection, species identification, and evaluation of vector control interventions. In conclusion, WINSA was created to enable data sharing and foster collaborative research in South America, with the aim of strengthening entomological surveillance and improving vector control, in line with WHO’s Global Plan for Insecticide Resistance Management [36] and the Global Vector Control Response [37]. Strengthening regional expertise and experience in vector resistance research will be highly beneficial for national programs to improve the monitoring and management of insecticide resistance. By fostering cross-border collaborations and partnerships, the WINSA will contribute to strengthening South America’s response to insecticide resistance and reducing its public health impact, contributing to regional efforts for controlling and eliminating certain VBDs by 2030. The network will also collaborate closely with national institutions, international organizations and key regional stakeholders involved in VBD control and prevention, to enable proactive VBD prevention programs and preparedness for rapid responses to outbreaks.
